# The use of a *Cissus quadrangularis/Irvingia gabonensis *combination in the management of weight loss: a double-blind placebo-controlled study

**DOI:** 10.1186/1476-511X-7-12

**Published:** 2008-03-31

**Authors:** Julius E Oben, Judith L Ngondi, Claudia N Momo, Gabriel A Agbor, Caroline S Makamto Sobgui

**Affiliations:** 1 Laboratory of Nutrition and Nutritional Biochemistry, Department of Biochemistry, B.P. 812, University of Yaoundé 1, Yaoundé, Cameroon; 2 CRPMT, Institute of Medicinal Plants Studies, Yaoundé, Cameroon

## Abstract

**Aim:**

To evaluate the effects of two formulations, *Cissus quadrangularis*-only and a *Cissus quadrangularis/Irvingia gabonensis *combination, on weight loss in overweight and obese human subjects.

**Methods:**

The study was a 10 week randomized, double-blind, placebo-controlled design involving 72 obese or overweight participants (45.8% male; 54.2% female; ages 21–44; mean age = 29.3). The participants were randomly divided into three equal (n = 24) groups: placebo, *Cissus quadrangularis*-only, and *Cissus quadrangularis/Irvingia gabonensis *combination. Capsules containing the placebo or active formulations were administered twice daily before meals; no major dietary changes nor exercises were suggested during the study. A total of six anthropomorphic and serological measurements (body weight, body fat, waist size; total plasma cholesterol, LDL cholesterol, fasting blood glucose level) were taken at baseline and at 4, 8 and 10 weeks.

**Results:**

Compared to the placebo group, the two active groups showed a statistically significant difference on all six variables by week 10. The magnitude of the differences was noticeable by week 4 and continued to increase over the trial period.

**Conclusion:**

Although the *Cissus quadrangularis-*only group showed significant reductions on all variables compared to the placebo group, the *Cissus quadrangularis*/*Irvingia gabonensis *combination resulted in even larger reductions. This apparently synergistic formulation should prove helpful in the management of obesity and its related complications.

## Background

The percentage of persons whose body weight is considerably greater than ideal continues to rapidly increase, particularly in the developed countries. As early as 1999–2000, approximately two-thirds of the U.S. adult population was classified as overweight or obese [1]. Excess body weight is one of the most important risk factors for all-cause morbidity and mortality; i.e., the likelihood of developing such conditions as Type 2 diabetes, heart disease, cancer, and osteoarthritis of weight-bearing joints increases with body weight [2-4]. In addition to individual pain and suffering, these conditions lead to substantial economic costs in national healthcare budgets [5].

Among the many factors responsible for overweight and obesity is the continuing decline in physical activity [5,6]. hence the probability of compliance with conventional weight-management programs, which often include increasing energy expenditure via physical activity, is low. It is not at all surprising to see the marketing of many new dietary slimming aids aimed at satisfying the need for palatable (as well as safe, effective, and therapeutic) options. In accord with this approach are numerous investigations of the effectiveness of medicinal plants as natural supplements in reducing body weight, e.g., *Cissus quadrangularis *(Linn) and *Irvingia gabonensis *(Aubry-Lecomte ex O'Rorke).

*Cissus quadrangularis *(CQ), a succulent vine native to West Africa and Southeast Asia, has been used in traditional African and Ayurvedic medicine for more than a century. Although some studies [7-10] have examined other uses of CQ, its role in fighting obesity and symptoms of metabolic syndrome has attracted interest in other parts of the world [11-14]. The unique chemical constituents of CQ–novel flavonoids and indanes, as well as phytosterols and keto-steroids–have shown promise as powerful and efficient antioxidants [13,14]. They also appear efficient for lipase and amylase inhibition, thereby providing a mechanism for weight loss via reduced oxidative stress, dietary fat, and carbohydrate blocking.

Researchers and therapy formulation experts have also tried to improve the properties of CQ by combining it with different ingredients (cf. Cylaris™, which contains chromium, selenium, green tea extract, etc.) Another potentially synergistic substance–*Irvingia gabonensis *(IG)–belongs to the *Irvingiaceae *family. The Irvingia tree, commonly known as bush mango, dikanut or African mango [15], is indigenous to West Africa. Although the flesh of the IG fruit is widely consumed, its most important part is the kernel which (in its fresh or dried form) is used to add flavouring and consistency to many dishes [16].

IG contains 50% fat, 26.4% total carbohydrate, 2.3% ash, 7.5% crude protein, and 14% fibre [16]. The high soluble fibre content effects the lowering of plasma cholesterol, triglycerides, and glucose concentrations. More importantly, the glycoproteins in the IG seeds seem to inhibit hydrolase enzymes by blocking the active sites or altering enzyme configuration. Recent investigation of the α-amylase inhibitory activity provides a mechanistic hypothesis for the numerous studies supporting IG's anti-diabetic potential via its ability to reduce fasting blood glucose levels [17].

In addition to their anti-diabetic activity, IG seeds have shown promise as an anti-obesity agent. A 2005 randomized, double-blind, placebo-controlled study reported significant differences between the IG treatment and placebo groups in weight and fat loss, as well as reductions in hip and waist circumference [18].

Alpha-amylase inhibitors are drug-design targets in the development of compounds for the treatment of diabetes, obesity and hyperlipaemia [19]. Although much research has focused on glycosidase inhibitors to control hyperglycemia, many forms of starch are digested as rapidly as glucose absorption [20,21]. Slowing the digestion and breakdown of starch has beneficial effects on insulin resistance and glycemic index control in people with obesity-related diabetes [20,22].

The present study was primarily designed to test the efficacy of a combination of these two extraordinary plants–*Cissus quadrangularis *and *Irvingia gabonensis*–in the management of obesity and obesity-related complications in humans.

## Methods

### Participants

Seventy-two obese or overweight subjects were recruited for the 10-week study. Based on physical examination and laboratory screening tests, all diabetics as well as pregnant and lactating women were excluded. None of the participants took any weight-reducing medication nor followed any specific diet for the duration of the trial period.

Of the 72 subjects, 33 (45.8%) were male and 39 (54.2%) were female. The mean BMI was > 26 kg/m, and the age range was 21–44 (mean age = 29.3).

The purpose, nature and potential risks of the study were explained to the patients, and all gave their written informed consent before participation. The Cameroon National Ethics Committee approved the protocol. The study was conducted in accordance with the Helsinki Declaration (1983 version).

### Study design/Intervention

The study was a randomized, double-blind, placebo-controlled design. The participants were randomly divided into three equal groups (n = 24): placebo; CQ-only extract, and CQ-IG combination. The placebo (250 mg) or active formulations (150 mg CQ and 250 mg CQ-IG) were administered twice daily before meals with 8–10 oz. of water. Since the capsules were identical in shape, colour and appearance, neither the participants nor the researchers knew which treatment was administered. The CQ and IG were proprietary extracts standardized to 2.5% keto-steroids for CQ (CQR-300) and 7% albumins for IG (IGOB131). All testing materials were supplied by Gateway Health Alliances, Inc., Fairfield, California, USA.

The 72 subjects were examined once a week during the 10-week study period, and their body weight, percent body fat, and waist circumference were recorded. Fasting blood samples were taken at baseline and at 4, 8, and 10 weeks. In addition to these physiological measurements, the patients' subjective impressions of their well-being (e.g., increased/decreased appetite, dizziness, gastrointestinal pains, etc.) were solicited and recorded at every visit. Although no major dietary changes or exercises were suggested, the subjects were queried re their physical activity and food intake.

### Anthropometric measurements

Body weight, percent body fat, and waist circumference were assessed at each visit with a Tanita™ BC-418 Segmental Body Composition Analyzer/Scale that uses bio-electrical impedance analysis for body composition analysis. Height was measured with a Harpended™ stadiometer, which measures the length of curved line staffage to the nearest 0.5 cm. Participants (12 hour fasted) were encouraged to wear light clothing before measurements were taken. The waist circumference was measured by soft, non-stretchable plastic tape on the narrowest and widest parts of the trunk.

### Serological/Laboratory methods

Blood samples were collected into heparinized tubes after a 12-hour overnight fast at the beginning of the study and after 4, 8, and 10 weeks of treatment. The concentrations of total cholesterol, LDL cholesterol, and fasting blood glucose in plasma were measured using commercial diagnostic kits from SIGMA Diagnostics, St. Louis, Missouri USA.

### Statistical Analysis

The data for each parameter was summarized (n, mean, and standard deviation) for Week 0 (Initial) and Weeks 4, 8, and 10 and for the intra-group percent differences (Initial vs. Week 4, Week 8, and Week 10). The percent change from baseline was tested for differences using the Mixed Effects Model, which is a flexible tool for analyzing longitudinal and repeated treatments. For each parameter, several measurements were made. This continuous measurement over a specified period justifies the sample size used for each group.

## Results

### Anthropomorphic characteristics (body weight, body fat, waist size)

As shown in Tables 1, 2, and 3, the two treatment (vs. placebo) groups showed a noticeable decrease in these three variables by week 4 and maintained–indeed, significantly increased–the magnitude of the differences throughout the 10-week trial period. By week 10, the differences in measures of body weight, body fat, and waist size between the CQ-IG combination group vs. the CQ-only and placebo groups were all statistically significant.

**Table 1:** Body weight: effectiveness of treatments

	**Body weight (mean kg)**				**Weight change (%)**		
	**Initial**	**4 weeks**	**8 weeks**	**10 weeks**	**4 Weeks-Initial**	**8 Weeks-Initial**	**10 Weeks-Initial**

**Placebo**	98.05 ± 12.30	98.76 ± 8.20	96.74 ± 10.60	95.99 ± 15.20	0.72	-1.33	-2.10
**CQ**	98.92 ± 10.60	95.77 ± 12.32^a^	91.47 ± 8.69^a^	90.19 ± 7.60^b^	-3.19	-7.53^†^	-8.82^†^
**CQ-IG**	99.79 ± 13.50	95.77 ± 7.40	90.91 ± 5.72^b ^*	87.95 ± 3.17^c ^**	-4.02^†^	-8.90^†^	-11.86^†^

^a^p < 0.05; ^b^p < 0.001; ^c^p < 0.0001 compared with **Placebo**

*p < 0.05; **p < 0.001; ***p < 0.0001 compared with **CQ**

^†^p < 0.05; ^‡^p < 0.001 compared with **Initial**; intra-group analysis

**Table 2:** Body fat: effectiveness of treatments

	**Body fat (mean %)**				**Fat reduction (**%**)**		
	**Initial**	**4 weeks**	**8 weeks**	**10 weeks**	**4 Weeks-Initial**	**8 Weeks-Initial**	**10 Weeks-Initial**

**Placebo**	33.32 ± 7.60	32.37 ± 12.86	32.31 ± 10.91	32.00 ± 14.63	-2.85	-3.33	-3.97
**CQ**	33.07 ± 10.26	30.81 ± 5.92	29.42 ± 5.49	28.23 ± 6.12^a^	-6.83	-11.05^†^	-14.63^†^
**CQ-IG**	35.66 ± 12.27	32.41 ± 7.91	29.53 ± 5.15	28.51 ± 4.17^a ^*	-9.11^†^	-17.19^†^	-20.06^‡^

^a^p < 0.05; ^b^p < 0.001; ^c^p < 0.0001 compared with **Placebo**

*p < 0.05; **p < 0.001; ***p < 0.0001 compared with **CQ**

^†^p < 0.05; ^‡^p < 0.001 compared with **Initial**; intra-group analysis

**Table 3:** Waist size: effectiveness of treatments

	**Waist (mean cm)**				**Waist Change (**%**)**		
	**Initial**	**4 weeks**	**8 weeks**	**10 weeks**	**4 Weeks-Initial**	**8 Weeks-Initial**	**10 Weeks-Initial**

**Placebo**	102.40 ± 16.26	101.82 ± 12.21	101.76 ± 13.30	101.37 ± 16.55	-0.56	-0.63	-1.00
**CQ**	99.83 ± 13.38	97.10 ± 18.57	93.81 ± 10.70^a^	91.20 ± 7.6^b^	-2.73	-6.03^†^	-8.64^†^
**CQ-IG**	104.30 ± 23.10	98.28 ± 17.41	96.00 ± 12.20^b ^**	82.42 ± 3.88^c ^**	-5.77^†^	-7.96^†^	-20.98^†^

^a^p < 0.05; ^b^p < 0.001; ^c^p < 0.0001 compared with **Placebo**

*p < 0.05; **p < 0.001; ***p < 0.0001 compared with **CQ**

^†^p < 0.05; ^‡^p < 0.001 compared with **Initial**; intra-group analysis

#### Body weight (Table 1)

Although the placebo group showed no change in body weight, the CQ-IG group lost 4 kg (4.0%) after just 4 weeks of treatment. To translate the difference in amounts lost to final outcomes, the 10-week mean body weight of the CQ-IG group was 88.0 kg vs 96.0 kg for the placebo group (p < 0.0001) and 90.2 kg for the CQ group (p < 0.001). In terms of intra-group mean % change from baseline to 10 weeks, the placebo, CQ and CQ-IG combo groups lost 2.1%, 8.8% (p < 0.05), and 11.9% (p < 0.05), respectively.

#### Body fat (Table 2)

As with body weight, the placebo group showed no significant change in %-body fat after 4 weeks whereas the CQ-IG group lost 3.2% body fat (i.e., a 9.11% reduction). To translate the differences in amount lost to final outcomes, the 10-week mean %-body fat of the CQ-IG group was 28.5% vs 32.0% for the placebo group (p < 0.05) and 28.2% for the CQ group (p < 0.05). In terms of intra-group mean % change from baseline to 10 weeks, the placebo, CQ and CQ-IG combo groups lost 4.0%, 14.6% (p < 0.05), and 20.0% (p < 0.001), respectively.

#### Waist size (Table 3)

Waist circumference is one of the most important determinants in the diagnosis of obesity and metabolic syndrome. Once again, whereas the placebo group showed a minimal decrease (0.6 cm or .6%), the CQ-IG group lost 6.0 cm (5.8%) after 4 weeks. By week 10, the reduction in waist size was 1.0 cm for the placebo group vs. 21.9 cm for the CQ-IG group.

To translate the difference in amounts lost to final outcomes, the 10-week mean waist size (cm) of the CQ-IG group was 82.4 cm vs 101.4 cm for the placebo group (p < 0.0001) and 91.2 cm for the CQ group (p < 0.001). In terms of intra-group mean % change from baseline to 10 weeks, the placebo, CQ and CQ-IG combo groups lost 1.0%, 8.6% (p < 0.05), and 21.0% (p < 0.05), respectively.

### Serological characteristics (total cholesterol, LDL cholesterol, fasting blood glucose)

As shown in Tables 4, 5, and 6, the treatment (vs. placebo) groups showed a noticeable decrease in these three variables by week 4. As was the case with the three anthropomorphic variables, the magnitude of the losses increased significantly over the duration of the 10-week trial period.

**Table 4:** Plasma total cholesterol level: effectiveness of treatments

	**Total cholesterol (mean mg/dL)**				**Change (**%**)**		
	**Initial**	**4 weeks**	**8 weeks**	**10 weeks**	**4 Weeks-Initial**	**8 Weeks-Initial**	**10 Weeks-Initial**

**Placebo**	146.20 ± 38.14	140.40 ± 11.11	150.06 ± 13.20	149.47 ± 19.16	-3.965	2.64	2.23
**CQ**	150.34 ± 21.24	122.31 ± 15.56^b^	116.40 ± 17.30^b^	110.21 ± 9.34^b^	-18.64^†^	-22.57^†^	-26.69^†^
**CQ-IG**	153.21 ± 20.21	108.45 ± 18.21^b ^**	89.48 ± 10.66^b ^***	85.33 ± 7.80^b ^***	-29.21^†^	-41.59^†^	-44.30^†^

^a^p < 0.05; ^b^p < 0.001; ^c^p < 0.0001 compared with **Placebo**

*p < 0.05; **p < 0.001; ***p < 0.0001 compared with **CQ**

^†^p < 0.05; ^‡^p < 0.001 compared with **Initial**; intra-group analysis

**Table 5:** Plasma LDL cholesterol level: effectiveness of treatments

	**LDL cholesterol (mean mg/dL)**				**Change (%)**		
	**Initial**	**4 weeks**	**8 weeks**	**10 weeks**	**4 Weeks-Initial**	**8 Weeks-Initial**	**10 Weeks-Initial**

**Placebo**	76.13 ± 8.02	79.47 ± 7.50	74.35 ± 9.02	73.87 ± 8.44	4.38	-2.34	-2.96
**CQ**	80.41 ± 8.30	66.30 ± 11.06^b^	63.69 ± 8.79^b^	64.20 ± 11.13^b^	-17.55^†^	-20.78^‡^	-20.16^‡^
**CQ-IG**	86.11 ± 7.82	60.30 ± 9.39^b ^**	57.28 ± 8.36^b ^**	44.18 ± 10.02^a ^**	-29.96^†^	-33.48^‡^	-48.69^‡^

^a^p < 0.05; ^b^p < 0.001; ^c^p < 0.0001 compared with **Placebo**

*p < 0.05; **p < 0.001; ***p < 0.0001 compared with **CQ**

^†^p < 0.05; ^‡^p < 0.001 compared with **Initial**; intra-group analysis

**Table 6:** Fasting blood glucose levels: effectiveness of treatments

	**Blood glucose (mean mg/dL)**				**Change (**%**)**		
	**Initial**	**4 weeks**	**8 weeks**	**10 weeks**	**4 Weeks-Initial**	**8 Weeks-Initial**	**10 Weeks-Initial**

**Placebo**	79.43 ± 11.63	78.34 ± 10.41	76.53 ± 10.42	77.32 ± 8.90	-1.37	-3.67	-2.65
**CQ**	80.32 ± 8.45	71.56 ± 5.28^a^	70.30 ± 9.40^a^	68.38 ± 7.78^b^	-10.90^†^	-12.47^†^	-14.85^†^
**CQ-IG**	87.68 ± 6.32	68.32 ± 11.11^b ^*	65.47 ± 8.31^b ^*	60.11 ± 4.31^b ^**	-22.07^†^	-25.32^†^	-31.44^‡^

^a^p < 0.05; ^b^p < 0.001; ^c^p < 0.0001 compared with **Placebo**

*p < 0.05; **p < 0.001; ***p < 0.0001 compared with **CQ**

^†^p < 0.05; ^‡^p < 0.001 compared with **Initial**; intra-group analysis

#### Plasma total cholesterol level (Table 4)

Although the placebo group showed a small, short-lived decrease (5.8 mg/dL) by week 4, the reduction for the CQ-IG group was 44.8 mg/dL (29.2%). To translate the difference in amounts decreased to final outcomes, the 10-week mean total cholesterol level of the CQ-IG group was 85.3 mg/dL vs 149.5 mg/dL for the placebo group (p < 0.001) and 110.2 mg/dL for the CQ group (p < 0.0001). In terms of intra-group mean % change from baseline to 10 weeks, the placebo group increased by 2.2%; the CQ and CQ-IG combo group decreased by 27.0% (p < 0.05) and 44.3% (p < 0.05), respectively.

#### Plasma LDL cholesterol level (Table 5)

In contrast to the small (3.3 mg/dL) increase in LDL shown by the placebo group by week 4, the CQ-IG group showed a 25.8 mg/dL (30.0%) decrease. To translate the difference in amounts decreased to final outcomes, the 10-week mean LDL cholesterol level of the CQ-IG group was 44.2 mg/dL vs 73.9 mg/dL for the placebo group (p < 0.05) and 64.2 mg/dL for the CQ group (p < 0.001). In terms of intra-group mean % change from baseline to 10 weeks, the placebo, CQ and CQ-IG combo groups decreased by 3.0%, 20.2% (p < 0.001), and 48.7% (p < 0.001), respectively.

#### Fasting blood glucose levels (Table 6)

In contrast to the small (1.1 mg/dL) decrease in blood glucose level shown by the placebo group by week 4, the CQ-IG group showed a 19.4 mg/dL decrease (22.1%). To translate the difference in amounts decreased to final outcomes, the 10-week mean blood glucose level of the CQ-IG group was 60.1 mg/dL vs 77.3 mg/dL for the placebo group (p < 0.001) and 68.4 mg/dL for the CQ group (p < 0.001). In terms of intra-group mean % change from baseline to 10 weeks, the placebo, CQ and CQ-IG combo groups decreased by 2.6%, 14.8% (p < 0.05), and 31.4% (p < 0.001), respectively.

### Adverse events

Adverse events with an incidence >3 included headache (4), lack of sleep (4), and gas (5). Since the incidence of all reported side effects was observed in the placebo group as well as in the treatment groups, it is probably safe to conclude that the CQ-IG formulation had few, if any, negative side effects.

## Discussion

It is generally accepted that slowing the spread of obesity (and its concomitant complications) requires a multi-dimensional approach including, perhaps, the use of novel treatments involving control at different levels; e.g. lipid metabolism, carbohydrate metabolism, satiety, etc. The present study showed that a therapy comprising a combination of different active substances had considerable potential.

In previous studies [12,13], the CQ-only formulation showed beneficial effects on weight as well as on parameters of metabolic syndrome. Oxidative stress resulting from obesity and metabolic syndrome seems to be gaining ground as a major factor in the development and progression of these conditions. When calorie intake exceeds energy expenditure, the substrate-induced increase in citric acid cycle activity generates an excess of mitochondrial NADH and reactive oxygen species [23].

Independent of the above, escalation of adipose tissue effects an increase in the secretion of inflammatory cytokines, interleukin-6, and tumor necrosis factor-alpha, which could result in increased circulating levels of C-reactive protein, inflammation, and cardiovascular disease [24]. Reducing the oxidative stress by CQ and/or IG ***[independently submitted for publication] ***could improve insulin sensitivity, inflammation, body-mass index, cardiovascular disease and related conditions.

Our results showed that the combination of CQ and IG had a synergistic effect on the reduction of total cholesterol, LDL-cholesterol, and fasting blood glucose when compared to CQ-only, thus creating a better anti-atherogenic agent. The efficacy of CQ has been linked to its content of various steroidal principles, a novel flavonoid (3-*O-*alpha-L-rhamnopyranosylkaempferol) and stilbene (3-(4-hydroxybenzylidene)-2-(2,5-dihydroxyphenyl)-1-(4-hydroxyphenyl)indane-4,6-diol), as well as four known structurally related flavonoids, and one stilbene [14]. These components also have the ability to inhibit certain enzymes like alpha amylase, glucosidase and lipase [11,14]. IG seeds, on the other hand, have been shown to have hypocholesterolemic, hypoglycaemic, anti-amylase, anti-lipase, and anti-oxidant properties [18,25].

A formulation comprising a combination of these two plant materials suggests one distinct possibility in the multi-dimensional management of obesity and its related complications.

## Authors' contributions

JO conceived, designed and coordinated the work, as well as prepared the manuscript. JN was involved in the co-design of the work as well as the draft of the manuscript. CM carried out analytical work, GA carried out analytical work and contributed in drafting the manuscript. CS carried out analytical and statistical analysis. All authors read and approved the final manuscript.
